# Multimodal evaluation of the pitchfork sign in a patient with type 2 macular neovascularization that was treated with aflibercept

**DOI:** 10.5935/0004-2749.2024-0148

**Published:** 2024-10-23

**Authors:** Isabela Spinelli Mota, Juliana Souza Gomes, Fernanda Cunha, Michelle Gantois

**Affiliations:** 1 Fundação Altino Ventura, Recife, PE, Brazil; 2 Hospital de Olhos de Pernambuco, Recife, PE, Brasil

**Figure 1:**
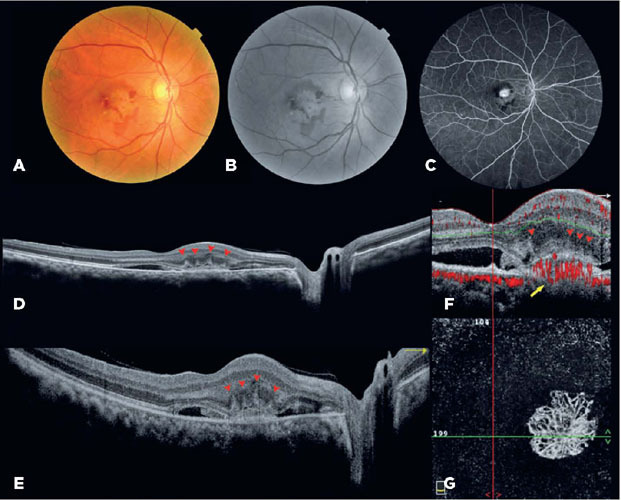
Multimodal images of the right eye obtained at the first visit. A) Retinography and B) redfree retinography showing subretinal hemorrhage in the macular region. C) Fluorescein angiogram showing leakage (hyperfluorescence). D) Optical Coherence Tomography B-scan (OCT-B) showing a pitchfork sign, choroidal thickening, and increased caliber of the Haller’s layer vessels. E) Finger-like vertical projections can be seen (red arrow head). F) OCTB showing pitchfork sign (red arrow head) and decorrelation signal (yellow arrow). G) . En-face OCTA showing a neovascular membrane in the outer retinal slab.

**Figure 2:**
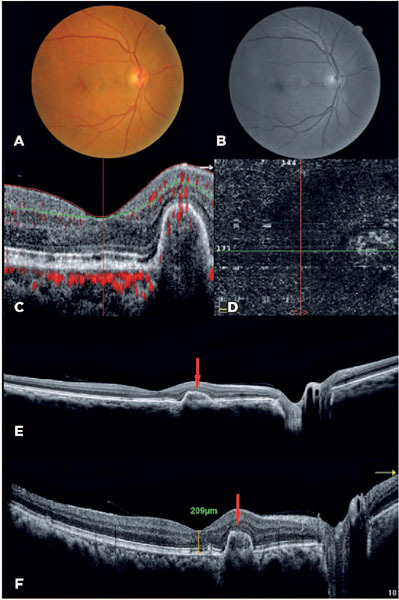
A) Retinography and B) redfree retinography showing remission of the retinal hemorrhage. C) Optical coherence tomography (OCT) angiography showing pigment epithelial detachment (PED) with a significantly reduced decorrelation signal. D) Involution of the neovascular formation can be seen in the external retinal slab. E and F) OCT B-scan showing the presence of PED and involution of the subretinal hyperre-flective material and subretinal fluid.

The pitchfork sign (PS) is a distinctive finding on optical coherence tomography (OCT) that is characteristic of type 2 macular neovascularization (MNV) secondary to punctate inner choroidopathy (PIC)^(1,2,3)^. A 57-year-old male presented to us with complaints of blurring of vision in the right eye (OD) for one month. At the time of admission, the best-corrected visual acuity was 20/150. OCT and OCT angiography revealed a PS (Figure 1). Thus, the patient was diagnosed with type 2 MNV secondary to PlC. A loading dose of three aflibercept intravitreal injections (1V1) was administered. No other drugs were prescribed. A good response was observed in the treated eye. At the 1-year follow-up, no neovascularization reactivity was observed, and the visual acuity had improved to 20/20 (Figure 2). The first-choice treatment for MNV is anti-VEGF IVIs. Other reports have described good morphofunctional results in patients treated with bevacizumab or ranibizumab. However, they reported the reappearance of new lesions^(2,4)^. In our patient, the visual acuity significantly improved and the retinal lesions regressed after the administration aflibercept 1V1s, without the use of corticosteroids or immunosuppressants.
